# Transcriptome Analysis on Maternal Separation Rats With Depression-Related Manifestations Ameliorated by Electroacupuncture

**DOI:** 10.3389/fnins.2019.00314

**Published:** 2019-04-05

**Authors:** Yuanjia Zheng, Jiang He, Lili Guo, Lin Yao, Xiaorong Zheng, Zhihua Yang, Yucen Xia, Xiaoli Wu, Yang Su, Nenggui Xu, Yongjun Chen

**Affiliations:** ^1^South China Research Center for Acupuncture and Moxibustion, Medical College of Acu-Moxi and Rehabilitation, Guangzhou University of Chinese Medicine, Guangzhou, China; ^2^School of Pharmaceutical Sciences, Guangzhou University of Chinese Medicine, Guangzhou, China; ^3^Center for Brain Science and Brain-Inspired Intelligence, Guangdong-Hong Kong-Macao Greater Bay Area, Guangzhou, China

**Keywords:** electroacupuncture, transcriptome, prefrontal cortex, maternal separation, depression

## Abstract

Maternal separation (MS), a stressful event in early life, has been linked to neuropsychiatric disorders later in life, especially depression. In this study we investigated whether treatment with electroacupuncture (EA) could ameliorate depression-related manifestations in adult animals that had adverse early life experiences. We demonstrated depression-like behavior deficiencies in a sucrose preference test and a forced swimming test in a rat model with neonatal MS. Repeated EA treatment at the acupoints Baihui (GV20) and Yintang (GV29) during adulthood was shown to be remarkably attenuated above behavioral deficits. Using unbiased genome-wide RNA sequencing to investigate alterations in the transcriptome of the prefrontal cortex (PFC), we explored the altered gene sets involved in circadian rhythm and neurotransmitter transporter activity in MS rats, and their expression tended to be reversed after EA treatment. In addition, we analyzed the interaction network of differentiated lncRNA– or circRNA–miRNA–mRNA by using the principle of competitive endogenous RNA (ceRNA). These results suggest that EA at GV20 and GV29 ameliorates depression-related manifestations by regulating the expression of multiple genes.

## Introduction

The mother–child relationship has been reshaped by rapid societal changes. Mother-newborn separation shortly after birth, for example, has become routine following hospital births (Csaszar-Nagy and Bokkon, 2018). In the past 30 years, the phenomenon of rural children living apart from their parents, who work in cities, has become so widespread that as many as 37.7% of children in China are affected (Wang, 2015). A growing body of literature indicates that adverse early life experience is significantly associated with susceptibility to stress-related psychopathologies such as depression (Gibb et al., 2007) and anxiety (Coplan et al., 2014; Ishikawa et al., 2015) disorders. Similarly, parental loss due to sudden death increases the vulnerability of children to depression (Brent et al., 2009). Although the molecular basis has not been fully elucidated, neonatal maternal separation (MS), an early-life adverse event, can have long-lasting effects on neural development and increases risk of adult psychopathology in human adults (Hanson et al., 2012).

Depression is a common mental disorder worldwide, with over 322 million people being diagnosed with it every year. As the leading cause of disability worldwide (Cai et al., 2015) participants with depression had greatly higher total healthcare costs than those without ($20,046 vs. $11,956; *p* < 0.01) in previous study (Unutzer et al., 2009). The selective serotonin reuptake inhibitors (SSRIs) and serotonin and noradrenaline reuptake inhibitors (SNRIs) that are currently the first-line treatment options for major depressive disorder (MDD) (Dale et al., 2015) usually require 4–6 weeks, and sometimes longer, to be effective (Liu et al., 2015). The side effects of their long-term use include sleep disturbance (Roohi-Azizi et al., 2018) and non-response to other classes of antidepressants (Li Q. et al., 2013). To induce a depression-like phenotype for investigating the antidepressant effects of the drugs, various rodent models were used, such as being exposed to chronic unpredictable mild stress, learned helplessness, chronic social defeat stress and so on (Cao et al., 2013; Alboni et al., 2017; Post and Warden, 2018). Adverse experience in early life is associated with stress-related psychopathologies, and previous studies have shown neonatal rats or mice exposed to MS were displayed depression-like behavior in adulthood (Vetulani, 2013; Sadeghi et al., 2016; Tchenio et al., 2017).

Acupuncture as a well-known useful treatment for depression has been proven. For example, acupuncture (electro- and manual) may moderately reduce the severity of depression by the end of treatment (SMD -0.66, 95% CI -1.06 to -0.25, five trials) in total 488 participants (Smith et al., 2010). According to a report from the Department of Veterans’ Affairs from Washington, DC, United States, depression is one of the nine clinical indications relevant to mental health for acupuncture (Hempel et al., 2014). However, the molecular mechanisms through which electroacupuncture (EA) modulates depressive behaviors are largely uncharacterized. Rat is an organism that provides a model with clinically relevant phenotypes for exploring new therapeutics (Jacob and Kwitek, 2002) and for studying the mechanism of acupuncture (Kou et al., 2017; Zhang Z.Y. et al., 2018). Furthermore, as acupuncture or EA can ameliorate depressive-like behaviors, a rat model of depression is a tool that has been widely used to investigate the antidepressant effects of acupuncture (Lu et al., 2016, 2017; Jiang et al., 2017). “Baihui” (GV 20) – “Yintang” (GV 29) are considered to be the optimized acupoint modules for mental illness (Duan et al., 2009a, 2014; Jiang et al., 2017), and our previous study indicates that EA at GV 20–GV 29 acupoints ameliorates cognitive deficits and improves hippocampal synaptic plasticity in adult rats with neonatal MS (Guo et al., 2018).

To investigate whether acupuncture can alleviate psychopathology in adults with stress-related adverse early life experiences, as well as to detail the mechanism by which acupuncture might regulate gene expression, we generated rat neonatal MS models and applied EA treatment at the GV20 and GV29 acupoints. Additionally, to explore experimentally the mechanisms involved with MS-induced depression, unbiased RNA sequencing (RNA-Seq) was used to identify rat genome-wide alterations in the prefrontal cortex (PFC) after MS and EA treatments. Furthermore, analysis of the interaction network of differentiated long non-coding RNA (lncRNA)- or circular RNA (circRNA)-miRNA-mRNA was performed using the principle of competitive endogenous RNA (ceRNA). Our study is the first to provide new information on the mechanism underlying anti-depressive effects of EA in adult rats with neonatal MS experience.

## Materials and Methods

### Experimental Animals

Male and nulliparous female Wistar rats of 180∼220 g were obtained from the Guangdong Medical Laboratory Animal Center. Animals were housed in the standard cages in controlled temperatures (20–22°C) and a 12-h light–dark cycle room. Food and water were available *ad libitum*. Animals entered the study at 8 weeks of age following a week-long acclimatization period and were mated at a 2:1 ratio of male to female rats. The female rats were raised alone once found to be pregnant. Wistar dams were assigned partly to control groups, with most to the molding groups. For each litter, the day of birth was named as postnatal day 0 (PND0) and the day after PND0 was the first day of molding, which was set as PND1. The experimental procedure was approved by the Animals Care and Use Committee of Guangzhou University of Traditional Chinese Medicine.

### Maternal Separation Model

From PND1 to PND21, MS that kept the mothers from their filial generation of molding groups into another cage for 4 h (9:00–13:00) was conducted every day. The mothers and litters in control groups were under no disturbance until weaning. All pups were weaned at PND21 and the males were housed four or five per cage until adult age, and the females were eliminated. The experimental procedure was approved by the Animals Care and Use Committee of Guangzhou University of Traditional Chinese Medicine. All efforts were made to minimize the animals’ suffering and to reduce the number of animals used for experiments.

### Animal Groups

#### Vehi, MS+Vehi, and MS+Flu

To determine whether the MS model induced depression in adulthood, at PND60, the litters belonging to healthy reservation groups were assigned to Vehicle group (Vehi), and the molding rats were assigned randomly into two groups: vehicle group (MS+Vehi) and Fluoxetine group (MS+Flu).

#### Cont, MS, EA, and Sham-EA

To examine whether the EA stimulation had effect on MS rats, in another trial, at the PND60, the litters belonging to healthy reservation groups were assigned to control group (Cont), and the molding rats were assigned randomly into three groups: maternal separation group (MS), MS with electro-acupuncture treatment group (EA) and MS with sham EA treatment group (Sham-EA).

### Treatment

All the treatments were performed from PND61 to PND81 every other day.

#### EA Stimulation

Using isoflurane (RWD, Shenzhen, China), the EA rats were positioned in the induction case with an anesthetized concentration of 5% for the initial 5 min for deep anesthetized condition, and then they were moved to the EA operating platform with a 2% concentration for the middle 10 min, decreasing to 1.5% for the last 5 min. Disposable acupuncture needles as previous reported (Guo et al., 2018) were inserted to the acupoints of GV20 and GV29. The Master-8 Stimulator (Master-8, AMPI, Israel) was connected to deliver electrical current to the needles. We set the output parameters as follows: holding the frequency constant at 2 Hz and intensities at 2 mA, for 15 min. EA stimulation was administered every other day for 15 min starting at 8:30 a.m. Rats in the Sham-EA group were anesthetized with isoflurane as the EA group. The difference was rats in the Sham-EA group received no electrical stimulation; a disposable acupuncture needle was attached to the surface near GV20 and GV29 but apart from any other known acupoints. The Cont rats and MS rats only received anesthetization conduction.

#### Fluoxetine Administration

The MS+Flu group were given injections of fluoxetine (10 mg kg^-1^, i.p.) while rats in the Vehi group and the MS+Vehi group received injections of an equal volume of saline (i.p.) from PND61 to PND81.

### Behavioral Tests

#### Body Weight Measurement

The body weights of rats in each group were measured every week during this period at 9 a.m. by balances (MS3002ts/00, Mettler Toledo).

#### Sucrose Preference Test (SPT)

In training days, each rat was exposure to two bottles of 1% sucrose solution for 24 h in the first day, and two bottles of tap water in the second day. In the third day, the bottles and food were withdrawn to make the rats hungry and thirsty. On the test day, bottle A contained 1% sucrose solution, and bottle B contained water. The fluid that was consumed from each bottle was measured after 24 h. The sucrose preference of each rat was calculated as 100 × [VolA/(VolA + VolB)], and the total fluid intake was calculated as VolA + VolB.

#### Forced Swimming Test (FST)

The FST was performed in a clear glass cylinder (height 45 cm, diameter 20 cm), which was filled to 30 cm with water (22–25°C). The test lasted for 5 min. The duration of immobility was recorded by JLBehv-FSR-4 (Shanghai Jiliang Software Technology Co., Ltd.).

#### Elevated-Plus-Maze Test (EPMT)

The elevated-plus-maze test consisted of two opposing open arms (50 cm × 15 cm) and two opposing enclosed arms (30 cm × 50 cm × 15 cm) that were connected by a central platform (15 cm × 15 cm), forming the shape of a plus sign. All of the measurements were taken in a dimly lit experimental room, in which the rats where acclimatized for at least 30 min before testing. The times that were spent in the open arms and the enclosed arms were recorded over a 10 min test period. The maze was cleaned with a solution of 20% ethanol in water between the sessions.

#### Open Field Test (OFT)

The open field presented an open box structure (80 cm × 80 cm × 40 cm) with a black square at the bottom. A camera device was installed directly above the central area of the open field. Before the experiment, the rats were conditioned for 60 min in advance in the test room. Uniform light and a quiet environment throughout the test were ensured. The rats were gently lowered into the central part of the square and allowed to move freely in the open field for 10 min. Its total distance and the time in the central area were recorded. The area was cleaned with 20% alcohol between the sessions. Only after the alcohol smell dissipated and the bottom of the box was without obvious signs of moisture were the rats tested.

#### Light-Dark Box Test

The test was carried out in a soundproof box with a light box (25 cm × 25 cm × 40 cm) and a dark box (25 cm × 25 cm × 40 cm). The dividing wall was inserted with an opening hole (8 cm × 7.5 cm) to allow the animal’s free movement from one compartment to another. The illumination was above the light box. The animal was released into the center of the light compartment (facing away from the opening) and was allowed to explore the area for 10 min. Distance traveled and time spent in different compartments were recorded by JLBehv-FSR-4 (Shanghai Jiliang Software Technology Co., Ltd.). The box was cleaned with a solution of 20% ethanol in water between the sessions.

### Radioimmunoassay

The reagent kits of corticosteroid (CORT) and adrenocorticotropic hormone (ACTH) for measurement of amounts were purchased from IZOTOP Institute of Isotopes Ltd. and Beijing North Biotechnology Research Institute, respectively. All determination procedures were according to the manufacturers’ instructions.

### Tissue Extraction and RNA Sequencing

Three samples per group (Cont, MS, and EA group) were sent for RNA sequencing. Rats were anesthetized with isoflurane (RWD, Shenzhen, China). The induced anesthesia concentration was 5% to perform tissue extraction. PFC was removed quickly and then put in liquid nitrogen for quick freezing. Then the PFC was stored at -80°C until tissue processing. Total RNA was extracted by Trizol reagent (Invitrogen) from tissue. RNA samples were prepared by using rRNA Depletion Kit (NEBNext^®^). The DNA libraries were applied to cluster generation and sequencing using cBot Operation for HD V2.5 Reagent and HiSeq X Operation for HD v2.5 reagent_v1.3 (Illumina). Sequencing was performed using Hiseq X ten (Illumina, United States). The raw data were deposited onto NCBI’s Read Gene Expression Omnibus (GEO) database and the accession number is GSE124387. RPKM (Reads Per kb per Million reads) was used to calculate gene expression from RNA-Seq data, which can eliminate the influence of gene length and sequencing amount for calculating gene expression. If multiple transcripts exist in a gene, we select the longest one to calculate sequencing depth and expression.

### Data Analysis

#### Statistical Analyses

All of the results are expressed as the means ± SEM. The statistical analyses were performed using SPSS 17.0 software. Potential differences between the mean values were evaluated using two-way or one-way analysis of variance (ANOVA) followed by the least significant difference (LSD) test for *post hoc* comparisons when equal variances were assumed. Non-parametric Kruskal–Wallis test was used to compare differences through groups when there was heterogeneity of variance. The significance level for all of the tests was set at *p* < 0.05.

#### Computational Analysis for RNA-Seq Data

Reads were filtered for quality by Fast-QC program and mapped to the rat genome (NCBI assembly Rnor_6.0) by the Hisat2 program. EB-Seq algorithm (Leng et al., 2013) was applied to filter the differentially expressed genes after the significant analysis and false discovery rate (FDR) analysis under the following criteria: fold change >1.5 or <0.667; FDR <0.05. According to the NCBI Gene Ontology database, GO analysis was performed by using Fisher’s exact test. It classifies the GO terms, and the FDR was calculated to correct the *p-value*. We utilized Miranda (Picao-Osorio et al., 2015) and RNAhybrid (Rehmsmeier et al., 2004) as the tools for predicting differentially expressed miRNA targets on circRNA, lncRNA, and mRNA.

For Series Cluster analysis, the raw expression values were converted into log2 ratio. Using a strategy for clustering short time-series gene expression data, we defined some unique profiles. Significant profiles have higher probability than expected by Fisher’s exact test and multiple comparison test (Ramoni et al., 2002). The following expression tendencies are what we had interest in: the genes decreased in MS rats compared to control rats but increase in EA rats contrasted with MS rats (decrease-increase type); the genes increased in MS rats compared to control rats but decreased in EA rats in contrast to MS rats (increase-decrease type). Based on that, the expression of mRNA and circRNA as well as mRNA and lncRNA satisfying this relationship are positively correlated; series cluster analysis is performed to identify a set of unique expression tendencies.

## Results

### Neonatal MS Induced Depression-Like Behavioral Deficits in Adults

**FIGURE 1:**
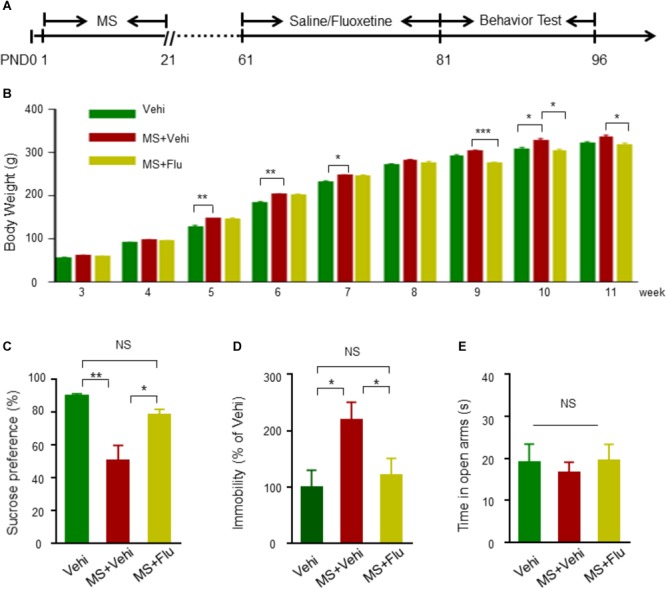
Neonatal MS induced depression-like behavioral deficits in adult Wistar rats. **(A)** The experimental schedule of MS, Fluoxetine administration, and behavioral test. Fluoxetine 10 mg kg^-1^ i.p. administration when rats were 8 weeks old before behavioral assessment at 11 weeks old. **(B)** Animals in all subgroups were weighted on the 3rd, 4th, 5th, 6th, 7th, 8th, 9th, 10th, and 11th postnatal weeks (*n* = 11 rats per group). **(C)** Reduced sucrose preference rate by rats with MS in SPT test compared to Vehi group rats, and the reduction was diminished after Fluoxetine administration (*n* = 11 rats per group, *F*_(2,30)_ = 30.82, one way-ANOVA). **(D)** Elevated immobility time in rats with MS+Vehi compared to Vehi rats and the elevation was ameliorated by Fluoxetine administration (*n* = 11 rats per group, *F*_(2,30)_ = 0.037, one way-ANOVA). **(E)** There was no difference on time in open arms in EMP test (*n* = 10–11 rats per group, *F*_(2,28)_ = 1.086, one way-ANOVA). Data are expressed as the means ± SEM. ^∗^*p* < 0.05, ^∗∗^*p* < 0.01, ^∗∗∗^*p* < 0.001. NS, not significant.

To confirm whether neonatal MS affects the onset of affective disorders in adults, we replicated the MS model to monitor depression- and anxiety-related behaviors in Wistar rats (Figure 1A). Before the beginning of the behavior test, body weight was measured every week. We found that the weight of rats with MS (MS+Vehi) increased slightly in adulthood compared with control (Vehi) and MS rats with Fluoxetine treatment (MS+Flu) (Figure 1B). In tests for depression-like behaviors, we applied SPT and FST, two well-known tests for detecting anhedonia and despair symptoms of depression disorder (Tye et al., 2013; Liu et al., 2018; Ji et al., 2019). Rats in the MS+Vehi group showed a significantly reduced sucrose consumption rate in SPT and increased duration of immobility in FST compared to Vehi. The administration of Fluoxetine restored sucrose consumption and immobility behavior deficits in both instances (Figure 1C,D). We next performed the EPM task, a well-established test of anxiety-like behavior (Walf and Frye, 2007). We found that rats from the three groups spent similar amounts of time in open arms (Figure 1E). To further detect whether MS rats have anxiety-like behaviors, the OFT and light/dark box test were performed (Bourin and Hascoet, 2003). There was no difference between the control and MS rats on the total distance, time in center in OFT, and time in light in light/dark box test (Supplementary Figure S1). Together, these results suggested that the MS model rat in the current study was successfully replicated for depression, but not anxiety.

### EA Induced Antidepressant-Like Effects

**FIGURE 2:**
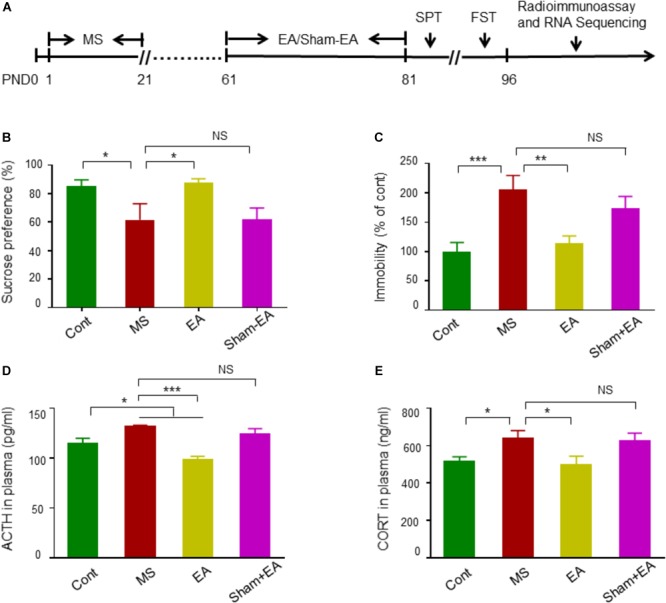
Electroacupuncture (EA) ameliorated depression-like behavioral deficits in rats exposed to neonatal MS. **(A)** The experimental schedule of MS, EA, or Sham-EA treatment, behavior tests and radioimmunoassay. **(B,C)** EA at GV20 and GV29 on rats with neonatal MS significantly elevated sucrose preference rate and reduced immobile rate in rats with neonatal MS (*n* = 10–13 rats per group, *F*_(3,44)_ = 7.949, non-parametric Kruskal–Wallis; *F*_(3,44)_ = 1.109, one way-ANOVA). **(D,E)** Radioimmunoassay of ACTH and CORT levels in plasma (*n* = 8–10 rats per groups, *F*_(3,32)_ = 2.884, one way-ANOVA; *F*_(3,36)_ = 0.793, one way-ANOVA). Data are expressed as the means ± SEM. ^∗^*p* < 0.05, ^∗∗^*p* < 0.01, ^∗∗∗^*p* < 0.001. NS, not significant.

To assess the effect of EA treatment on depression-like behavioral deficits in adult rats that suffered from MS, rats were tested for sucrose consumption in SPT and the total duration of immobility in FST (Figure 2A). Rats in the MS and the Sham-EA groups exhibited a decreased rate of sucrose consumption compared to controls (Figure 2B). However, the level of sucrose consumption in the MS+EA group was similar to the control group (*p* > 0.05), suggesting the anhedonia was attenuated after the EA treatment. Similarly, rats in the MS and Sham-EA groups showed increased immobility time compared to controls in FST. However, EA treatment dramatically decreased the total duration of immobility in rats with neonatal MS (Figure 2C). These results suggested that MS-induced depression-like behavior deficits were ameliorated after EA treatment.

To further demonstrate that EA could attenuate depression-like impairment in rats with neonatal MS, we measured the plasma ACTH and CORT levels, which is a biological indicator of depression and in part explains the relationship between hypothalamic-pituitary-adrenal (HPA) axis regulation and MS-induced depression (Mirescu et al., 2004; van der Doelen et al., 2014; Gururajan et al., 2016; Salvat-Pujol et al., 2017). Rats in the MS and Sham-EA groups showed dramatically increased plasma ACTH and CORT levels, which were significantly decreased by EA treatment (Figure 2D,E). These results indicated that repeated EA intervention induced antidepressant-like effects in rats with neonatal MS.

### RNA-Seq of PFC Transcriptome

We further investigated the possible mechanisms after confirming the anti-depressive effects of EA on rats with neonatal MS. RNA was extracted from the PFC, a brain area which is highly associated with the onset of depression (Kumar et al., 2015; Seo et al., 2017), in control, MS and EA therapy rats. After depletion of rRNA, unbiased deep sequencing was performed to an average depth of ∼89 million reads per sample in the three groups. Approximately 68 million reads mapped to unique locations on the NCBI assembly Rnor_6.0 reference genome (Supplementary Table S1).

### Identification of Differentially Expressed Genes Under EA Treatment in PFC of Rats With Neonatal MS

**FIGURE 3:**
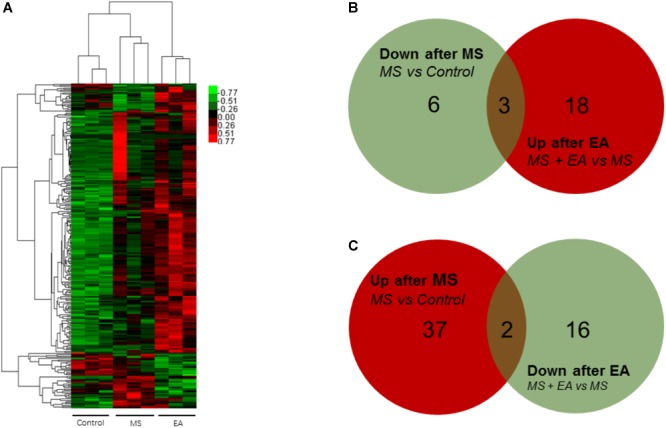
Differential mRNA expression in the prefrontal cortex of Cont, MS, and MS+EA in adult rats. **(A)** Heatmap of differentially expressed genes between Cont, MS, and MS+EA samples (*p* < 0.05) with green and red spectrum colors indicating downregulated and upregulated expression, respectively. **(B,C)** Venn diagrams show overlaps of differentially expressed genes between experimental groups. Three genes decreased in MS group but increased in EA group. Two genes increased expression in MS group but decreased in EA group.

To determine the regulation of mRNA expression, we performed an unsupervised clustering analysis of the significantly regulated genes in the PFC (Figure 3A). The EBseq algorithm was applied to filter the differentially expressed genes after the significant and FDR analyses (fold change >1.5 or <0.667; FDR <0.05). Forty-eight genes were significantly and differentially expressed in MS rats relative to control rats, with 39 upregulated genes and 9 downregulated genes. Thirty-nine genes in the EA rats relative to the MS rats show differential expression, with 21 genes upregulated and 18 genes downregulated (Supplementary Table S2). Venn analysis was additionally applied to learn the possible marker that participates in the exertion of acupuncture’s antidepressant effect. We found three of them (*Ucp3, Cplx3, Dbp*) can be reversed by EA treatment in the nine downregulated genes following MS. Similarly, EA treatment reversed 2 (*LOC102555167, Syt6*) of the 39 upregulated genes following MS (Figure 3B,C).

To show the possible cellular functions linked to differentially expressed genes, we used Gene Ontology (GO) enrichment analysis for the differentially expressed genes across three domains, including molecular functions (MF), cellular components (CC), and biological processes (BP) (Table 1). The genes that differentially decreased in the MS group but tended to be reversed after EA treatment were involved in synaptic vesicle exocytosis, circadian rhythm, syntaxin binding and synapse, and so on. Upregulated gene sets in the MS rats and reversed in the EA group were involved in mitochondrial transport, oxidative phosphorylation uncoupler activity, etc.

**Table 1:** The significant GO terms downregulated in MS but upregulated in EA or upregulated in MS but downregulated in EA (*p* < 0.05).

Domain	GO terms	
	Down-regulated in MS and Up-regulated in EA	Up-regulated in MS and Down-regulated in EA
BP:	Synaptic vesicle exocytosis	Response to superoxide
	Acrosomal vesicle exocytosis	Response to steroid hormone
	Regulation of neurotransmitter secretion	Mitochondrial transport
	Acrosome reaction	Response to glucocorticoid
	Insulin secretion	Cellular response to hormone stimulus
	Neurotransmitter transport	Response to activity
	Circadian rhythm	
	Rhythmic process	
MF:	Syntaxin binding	Hydrogen ion transmembrane transporter activity
	Calcium-dependent protein binding	Oxidative phosphorylation uncoupler activity
	Calcium-dependent phospholipid binding	
	Clathrin binding	
	SNARE binding	
	Neurotransmitter transporter activity	
	RNA polymerase II regulatory region sequence-specific DNA binding	
CC:	Nucleus	Mitochondrial membrane
	Synapse	
	Extrinsic component of membrane	
	Synaptic vesicle membrane	
	Perinuclear endoplasmic reticulum	

**FIGURE 4:**
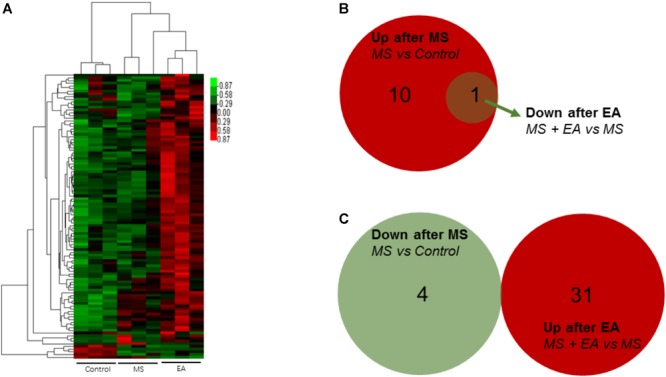
Differential lncRNA expression in the prefrontal cortex of Cont, MS, and MS+EA in adult rats. **(A)** Heatmap of differentially expressed lncRNAs between Cont, MS, and MS+EA samples (*p* < 0.05) with green and red spectrum colors indicating downregulated and upregulated expression, respectively. **(B,C)** Venn diagrams show overlaps of differentially expressed lncRNAs between experimental groups. One gene increased expression in MS group but decreased in EA group. No genes decreased expression in MS group but increased in EA group.

Long non-coding RNA is a type of RNA molecule that is more than 200 bp in length and has no protein coding ability. Using the same method as above, we found 11 genes increased significantly in the MS rats relative and 4 genes decreased relative to controls. Thirty-one genes increased in the EA rats relative to the MS rats while one gene decreased. Venn analysis shows that 1 gene co-occurred in two comparison groups: Rps2-ps2 (Figure 4 and Supplementary Table S2).

**FIGURE 5:**
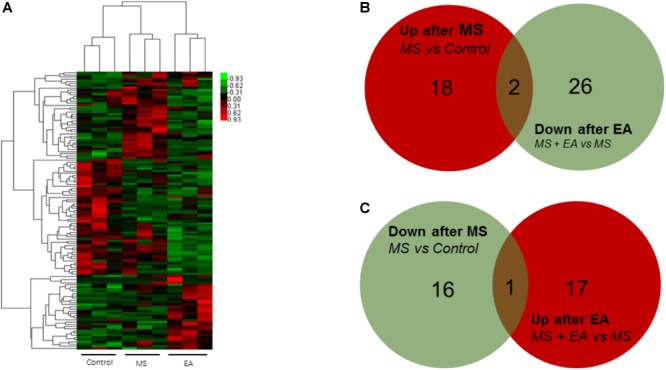
Differential circRNA expression in the prefrontal cortex of Cont, MS, and MS+EA in adult rats. **(A)** Heatmap of differentially expressed circRNAs between Cont, MS, and MS+EA samples (*p* < 0.05) with green and red spectrum colors indicating downregulated and upregulated expression, respectively. **(B,C)** Venn diagrams show overlaps of differentially expressed circRNAs between experimental groups. Two genes increased expression in MS group but decreased in EA group. One gene decreased expression in MS group but increased in EA group.

Circular RNA is a new class of RNA that differs from traditional linear RNA. It has a closed loop structure and is abundantly present in the eukaryotic transcriptome. CircRNAs were predicted by finding reads that cover both exons, but the direction is opposite to linear RNA. Twenty genes increased significantly in the MS rats relative to control rats and 17 genes decreased. Eighteen genes increased in the EA rats relative to the MS rats while 28 genes decreased. Venn Analysis shows that expression of two genes increased in the MS group but decreased in the EA group, while one gene decreased in the MS group but increased in the EA group. According to the position of the gene loop, these three genes were derived from *LOC102555866, Npepo, Cdh12* (Figure 5 and Supplementary Table S2).

**FIGURE 6:**
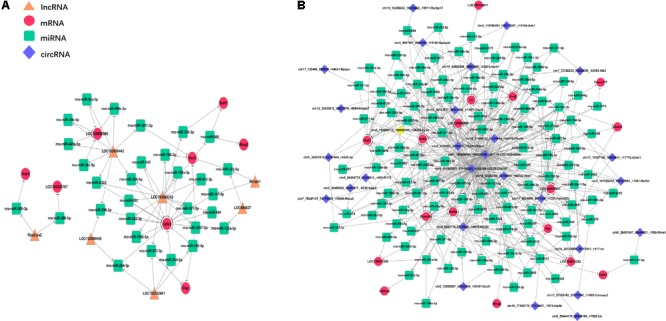
Interaction network of differentiated lncRNA–miRNA–mRNA and circRNA–miRNA–mRNA in the prefrontal cortex of Cont, MS, and MS+EA in adult rats. Diamond, round rectangle, ellipse, and triangle represent circRNA, miRNA, mRNA, and lncRNA, respectively. The genes shown in this figure are the same that belong to the increase-decrease type/decrease-increase type. **(A)** In the lncRNA–miRNA–mRNA predicted interaction network, 30 miRNAs are predicted to be possible target genes for regulating them. **(B)** In the circRNA–miRNA–mRNA predicted interaction network, 116 miRNAs are predicted.

MicroRNA (miRNAs) is a short-chain RNA about 22 nt in length that can reverse the expression of the target gene by inhibiting its translation or degrading it. Competitively binding miRNAs to regulate the expression of target genes is called competitive endogenous RNA (ceRNA). To explore the relationship between mRNA expression and its regulation, co-expression analysis of lncRNA/circRNA, miRNA and mRNA was carried out using the principle of ceRNA. First, the Miranda and the RNAhybrid algorithms were used to predict the target miRNAs for the circRNA, lncRNA, and mRNA, respectively. The combination of the two algorithms was used as the final result (Supplementary Table S3). Then, the genes with the same expression trend (both belong to decrease/increase – increase/decrease type) of lncRNA/circRNA and mRNA were extracted, and the lncRNA/circRNA, miRNA, and mRNA were combined to form an interaction network after Series Cluster analysis (Supplementary Table S3). In the lncRNA-miRNA-mRNA predicted interaction network, seven lncRNAs and eight mRNAs had the same expression trend (belonging to the increase-decrease type/decrease-increase type). Thirty miRNAs were predicted to be possible target genes for regulation (Figure 6A). In the circRNA-miRNA-mRNA predicted interaction network, 28 circRNAs and 17 mRNAs had the same trend. One hundred sixteen miRNAs were predicted to be potential target genes to regulate these circRNA and mRNA changes (Figure 6B).

## Discussion

Maternal separation rats exhibited a significantly lower preference for sucrose and higher immobility time (Figure 1C,D), but these behaviors were reversed in the fluoxetine injection group, suggesting MS is a stable and reliable means to model depression-like behaviors. These results are consistent with previous studies that showed chronic stress in early life could induce depressive-like behaviors in adults rats or mice (Arborelius et al., 2004; Vetulani, 2013; Masrour et al., 2018; Ruiz et al., 2018). However, some animal studies have shown that neonatal MS induced anxiety-like behaviors (Banqueri et al., 2017; Auth et al., 2018). Contrastingly, our result in the EPM task, OFT and Light-dark box test showed that rats with neonatal MS spent similar amounts of time in the open arms, center area, and light box, respectively, compared to controls (Figure 1E and Supplementary Figure S1). This difference may be due to a variation of species of animals (rats vs. mice), the MS protocol (longer vs. shorter duration of separation), and the different postnatal periods (PDN1-21 vs. PDN2-15) of MS (Auth et al., 2018). Meanwhile, the difference of housing conditions during the separation period (staying in home cage under 20–22°C vs. being kept in a new cage under 30°C) may also contribute to the variation of behavior phenotypes in adult MS (Banqueri et al., 2017). In addition, we found that the weight of rats with MS increased slightly in adulthood compared to control mice, which is consistent with previous studies (Macri et al., 2008; Yoo et al., 2013; Gonzalez-Pardo et al., 2019). However, other studies show normal body weight or reduced weight of MS rats in adulthood (Ryu et al., 2009; Paternain et al., 2016). Therefore, the relationship between body weight, located environment and MS-induced behavior deficits of animals still needs to be further studied.

Acupuncture (electro- and manual) is a treatment generally recommended for mental illnesses, including depression (Smith et al., 2010; Hempel et al., 2014). Recently, EA at the GV20 and GV29 acupoints has been reported to alleviate depression-related symptoms in model rats induced by chronic unpredictable mild stress (Duan et al., 2009b). Our results are in line with this finding, showing that EA significantly enhanced the sucrose uptake rate in the SPT and decreased the immobility duration in the FST, enabling the most characteristic presentations of depression to be reversed (Han et al., 2015). In addition, early-life stress influenced mature adults with sustained hyper-activation of the HPA axis (Maniam et al., 2014). Consistent with this conclusion, we found rats with MS induced a higher level of ACTH and CORT in their plasma compared to healthy rats (Figure 2D,E), which may in part reveal the potential relationship between early life adverse stress and depression in adulthood (Choi et al., 2018; Frost et al., 2018). Notably, the EA treatment can reverse the increased concentration of ACTH and CORT in rats with MS (Figure 2D,E), which is compatible with other studies showing that EA regulates the function of the HPA axis to treat depression (Tanahashi et al., 2016). Together, these results further confirmed that EA is an effective therapy for depression induced by neonatal MS.

Little is known of the mechanism of depression caused by MS, and the mechanism of anti-depression after EA. To the best of our knowledge, this was the first study to show EA has anti-depressive effects in a depression rat model induced by early MS. We detected the PFC genome-wide transcriptome of male rats suffering from MS to reveal the antidepressant effects of EA at the molecular level. Previous studies found EA can alter gene expression in a chronic unpredictable mild stress-induced rat model for mRNA or miRNA by using microarray or RNA-sequencing (Duan et al., 2014, 2016). However, instead of constructing the cDNA libraries by extracting poly-A (Wang et al., 2017), we constructed them after rRNA depletion to obtain data at a larger scale. Sequencing depth involves mRNA (Figure 3), lncRNA (Figure 4), and circRNA (Figure 5), which allowed us to detect differences in gene expression at the transcriptome level and predict the regulatory mechanisms that may mediate changes to their expression by ceRNA (Figure 6). These results suggest more comprehensive perspectives to explore the antidepressant mechanisms of acupuncture.

By GO enrichment analysis, we found altered gene sets are involved in circadian rhythm (Table 1), which is in accordance with other studies. From the dataset of transcripts from postmortem brains, it has been observed that >100 transcripts exhibited rhythmicity across six brain regions, including the dorsolateral PFC, while rhythmicity was much weaker in these brain areas of patients with MDD compared to those with no history of psychiatric disorders. Correspondingly, the canonical clock genes, including *Arntl, Per2-3*, and *Dbp* found in subject transcripts were observed in our analysis (Li J.Z. et al., 2013; Bunney et al., 2015). Furthermore, the clock genes (*Arntl, Per2-3*) of animals suffering from chronic mild stress (CMS) were strongly expressed in the PFC, but did not follow a circadian rhythm. The other study also found a reduction of the BMAL1 protein (*Arntl*) in the PFC of CMS rats (Calabrese et al., 2016; Christiansen et al., 2016). Consistent with our conclusions, one previous study reported EA can ameliorate the bowel dysfunction in spinal cord injury rats, and increase *Per2* expression (Cheng et al., 2016). Another reported that EA has regulatory effects on the circadian rhythm of temperature in CMS rats (Yao et al., 2014).

Additionally, we found the altered genes enrich in the neurotransmitter transporter activity, syntaxin binding, synaptic vesicle membrane. The genes corresponding to the GO terms are *Cplx3* and *Syt6*. *Cplx3* (complexin 3) is a subunit of the presynaptic protein that affects synaptic transmission. Previous studies indicated that *Cplx3* can affect the neurotransmitter release process by modulating exocytosis (Vaithianathan et al., 2015; Mortensen et al., 2016). Also, activity-dependent BDNF release via endocytic pathways can be regulated by syt6 and complexin (Wong et al., 2015), which is closely linked to depression (Gururajan et al., 2016). Thus, the above altered expression of the syntaxin binding may provide a means to investigate EA anti-depression effects.

Some previous studies suggested lncRNAs with mouse depression models may relate to depression pathologies (Bond et al., 2009; Huang et al., 2017). Furthermore, a study on peripheral blood profiling also found the expression of certain lncRNAs was changed in patients with MDD (Liu et al., 2014). In CMS mice, total saponins from the leaves of Panax notoginseng inhibited depression by regulating circRNA expression (Zhang H. et al., 2018). In the peripheral blood mononuclear cells of MDD patients, hsa_circRNA_103636 was significantly altered, suggesting that circRNA may be a potential novel biomarker (Cui et al., 2016). This suggests that lncRNA and circRNA play a non-negligible role in the mechanisms of pathological depression.

There are many examples of using transcriptome data analysis to investigate the mechanism of acupuncture without co-expression analysis, which could lead to the limitation of unclear and false positives targets (Fu et al., 2014; Hansen et al., 2014; Wang et al., 2015). Using ceRNA, we narrowed the targets of mRNA from 31 to 17 genes with a circRNA_miRNA_mRNA network, and narrowed the targets of mRNA from 31 to 8 genes with a lncRNA_miRNA_mRNA network (Figure 6), pointing the way for further studies to verify the genes and describe their function.

## Conclusion

In summary, our investigation indicates that attenuated neonatal MS induced depression-related manifestation by repeated EA treatment at the acupoints GV20 and GV29 during adulthood. Furthermore, we explored the altered gene sets involved in circadian rhythm and neurotransmitter transporter activity in MS rats through unbiased genome-wide RNA sequencing to investigate alterations in the transcriptome of the PFC.

## Ethics Statement

The experimental procedure was approved by the Animals Care and Use Committee of Guangzhou University of Traditional Chinese Medicine.

## Author Contributions

YC and NX designed the experiments. YZ and JH conducted the EA treatment and data analysis. ZY, XZ, and YS conducted the behavioral tests. YC and LG performed the radioimmunoassay analysis. YZ, JH, and YC wrote the manuscript. LY, XW, and YX helped revise the manuscript. All authors read and approved the final manuscript.

## Conflict of Interest Statement

The authors declare that the research was conducted in the absence of any commercial or financial relationships that could be construed as a potential conflict of interest.

## Supplementary Material

FIGURE S1 Results of OFT and light/dark box test between Cont and MS group. **(A,D)** Track graphs show the moving trail of Cont and MS rats in OFT and light/dark box test. **(B,C)** In the OFT, there was no difference in the total distance and time in center between two groups [*n* = 10–11 rats per groups, *F*(1,19) = 1.348, Student’s *t-test*; *F*(1,19) = 0.932, Student’s *t-test*]. **(E,F)** There was no difference in the total distance and time in light among control and MS rats in light/dark box test (*n* = 10–11 rats per groups, *F*(1,19) = 0.619, Student’s *t-test*; *F*(1,19) = 1.335, Student’s *t-test*).

TABLE S1 Statistical results by sequence quality control and the mapping rate by comparing with reference genes.

TABLE S2 Differentially expressed mRNA/lncRNA/circRNA in rats.

TABLE S3 Detail of the lncRNA/circRNA-miRNA-mRNA predicted interaction network.
