# Supplementary figures and images for: Transcriptome Analysis on Maternal Separation Rats With Depression-Related Manifestations Ameliorated by Electroacupuncture
Source: Front Neurosci. 2019 Apr 5;13:314. doi: 10.3389/fnins.2019.00314 (PMC6460510; doi:10.3389/fnins.2019.00314)

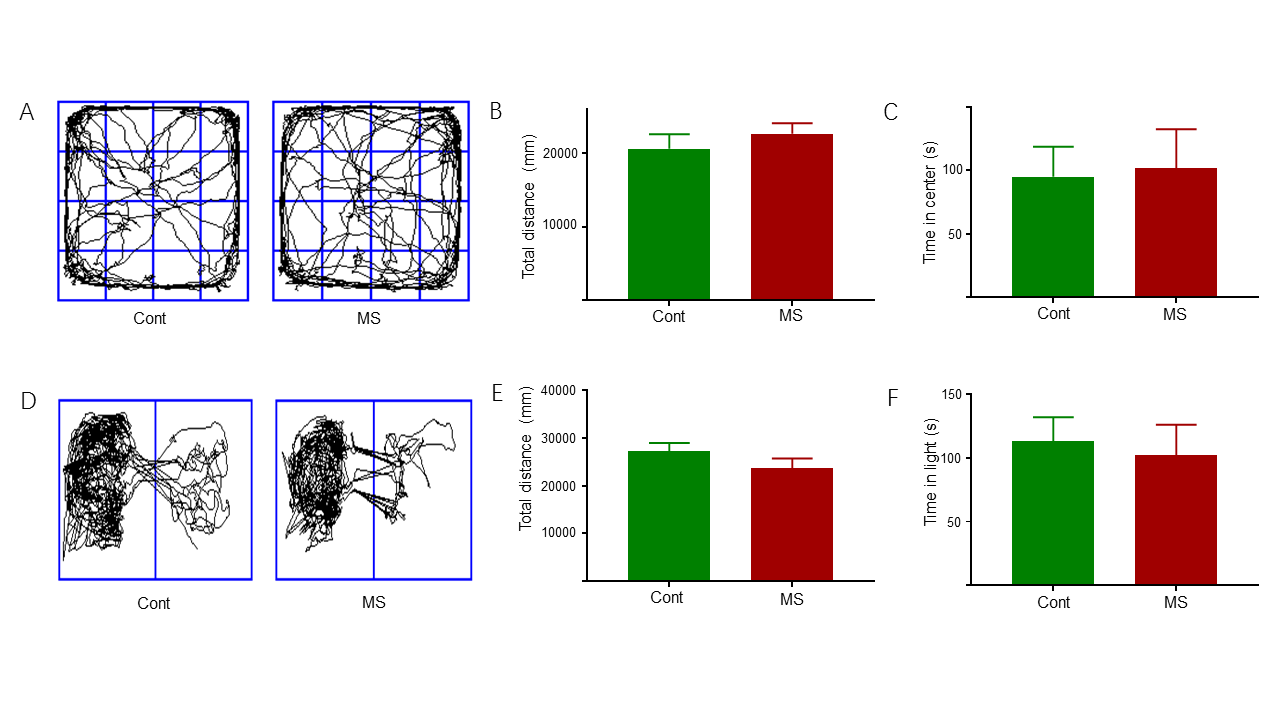

Supplement: FIGURE S1 — Results of OFT and light/dark box test between Cont and MS group. (A,D) Track graphs show the moving trail of Cont and MS rats in OFT and light/dark box test. (B,C) In the OFT, there was no difference in the total distance and time in center between two groups [n = 10–11 rats per groups, F(1,19) = 1.348, Student’s t-test; F(1,19) = 0.932, Student’s t-test]. (E,F) There was no difference in the total distance and time in light among control and MS rats in light/dark box test (n = 10–11 rats per groups, F(1,19) = 0.619, Student’s t-test; F(1,19) = 1.335, Student’s t-test). [file Image_1.TIF]
